# Biopolymer-Assisted Hydrothermal Synthesis of Manganese Cobalt Spinel Oxide (MnCo_2_O_4_) Using Cellulose and Chitosan for Enhanced Catalytic Performance

**DOI:** 10.3390/polym17233138

**Published:** 2025-11-25

**Authors:** Sultan Alhassan

**Affiliations:** Department of Physics, College of Science, Jouf University, Sakaka P.O. Box 2014, Saudi Arabia; ssalhassan@ju.edu.sa

**Keywords:** MnCo_2_O_4_, photocatalytic degradation, hydrothermal, methylene blue (MB), environmental remediation

## Abstract

This study reports the synthesis and photocatalytic performance of MnCo_2_O_4_ nanospinels fabricated with biodegradable stabilizers—chitosan (CHT) and biomass-cellulose (BC)—for the degradation of methylene blue (MB) under UV-rich solar irradiation. Structural and optical analyses (FTIR, XRD, XPS, PL, TGA, UV–Vis, and BET) revealed that the stabilizers significantly improved crystallinity, porosity, and charge-carrier separation while suppressing electron–hole recombination. Among the catalysts, MnCo_2_O_4_–CHT exhibited the best performance, achieving 96% MB degradation within 120 min, with the highest apparent rate constant (k_app_ = 0.0203 min^−1^) and shortest half-life (t_1_/_2_ = 34.2 min). In comparison, MnCo_2_O_4_–BC and MnCo_2_O_4_–Neat achieved 65% and 45% degradation, respectively. The enhanced activity of MnCo_2_O_4_–CHT is attributed to the chelation and electron-donating ability of chitosan’s –NH_2_ and –OH groups, which facilitate efficient charge transfer and reactive oxygen species (ROS) generation. These findings demonstrate the effectiveness of stabilizer-assisted synthesis in tuning the physicochemical properties of spinel oxides, offering a sustainable and high-performance photocatalyst for environmental remediation and wastewater treatment applications.

## 1. Introduction

Water contamination poses a severe threat to ecosystems and human health, driven by industrial discharges, agricultural runoff, and urbanization. The presence of organic dyes, heavy metals, and other toxic pollutants in wastewater has led to an urgent need for effective water remediation technologies. Among these pollutants, synthetic dyes such as methylene blue (MB) are particularly concerning due to their widespread use, resistance to biodegradation, and adverse environmental impacts. MB was chosen as the model pollutant due to its well-defined chemical structure, stability, and strong optical absorbance, which make it an ideal compound for evaluating photocatalytic performance and degradation kinetics. Although industrial wastewater contains complex mixtures of contaminants, MB is widely used as a standard model dye in photocatalytic studies because its chromophore allows for easy monitoring of degradation by UV–Vis spectroscopy. Furthermore, MB is a cationic thiazine dye commonly found in effluents from textile, paper, and pharmaceutical industries, representing a significant class of industrial pollutants. Its persistence, toxicity, and resistance to biodegradation make it environmentally relevant for assessing water treatment technologies. Using MB also enables comparative benchmarking of catalytic efficiency with previous studies, facilitating performance evaluation and optimization under controlled experimental conditions. Advanced water treatment methods have increasingly turned to photocatalysis as a promising solution. This technique uses light-activated catalysts to degrade and mineralize pollutants, offering an eco-friendly and efficient alternative to conventional methods [1,2,3]. In methylene blue (MB) photocatalytic degradation, noble metals like platinum, gold, palladium, and silver are effective cocatalysts but are limited by high cost and scarcity. Consequently, research now focuses on developing cost-effective, efficient, and durable alternatives capable of harnessing solar energy for sustainable water remediation [4,5,6].

Metal oxides have been widely studied as photocatalysts, but their efficiency is often limited by poor visible-light response and rapid charge recombination. Depositing cocatalysts to form surface heterojunctions effectively enhances charge separation, suppresses recombination, and creates active sites that lower reaction barriers, thereby improving photocatalytic performance [7,8,9,10,11,12]. Mixed-valent transition-metal oxides (MVMOs) have attracted attention as photocatalysts due to their cost-effective, unique structural properties, ability to harness light energy, and capacity to generate reactive oxygen species (ROS) upon light irradiation. These materials hold the potential to address the dual challenges of water remediation and sustainability by leveraging their high surface area, tunable band gaps, and reusability. MVMOs, such as ZnCo_2_O_4_, NiCo_2_O_4_, CuCo_2_O_4_, ZnAlTi, and other different composites, have demonstrated exceptional performance in degrading methylene blue and other organic contaminants in water. These catalysts utilize sunlight or UV light to break down complex pollutants into less harmful substances, with high efficiencies reaching under optimized conditions [13,14,15,16,17,18,19]. Through advanced material design, mixed-valent metal oxides (MVMOs) offer a sustainable route to efficient water remediation, with tailored compositions that enhance catalytic activity through synergistic interactions among multiple metal ions [20,21,22].

To address this, biopolymers such as chitosan (CHT) and biomass-derived cellulose (BC) are increasingly recognized as ideal stabilizers for nanocatalyst fabrication. Chitosan—a cationic polysaccharide derived from chitin—possesses abundant amino and hydroxyl functional groups that facilitate strong metal–biopolymer interactions, enhance nanoparticle dispersion, and improve photocatalytic stability [23]. Moreover, chitosan exhibits natural chelating properties, biocompatibility, and a high adsorption capacity for dyes due to its protonated amine groups [24]. Recent studies have demonstrated that chitosan composites with metal oxides such as ZnO, TiO_2_, and Ce-TiO_2_ achieve dye degradation efficiencies of 95–99%, outperforming pure oxides due to enhanced electron transport and reduced recombination [25,26]. Similarly, cellulose, the most abundant biopolymer on Earth, offers excellent mechanical stability, surface hydroxyl functionality, and hydrophilicity, which facilitate the formation of robust nanocomposites with controlled porosity and enhanced surface area [27]. In combination with chitosan, cellulose nanocrystals can further improve film homogeneity, water resistance, and charge carrier transport, providing a dual-function stabilizing and reinforcing matrix [28]. Compared with other biopolymers such as alginate, guar gum, or starch, chitosan and cellulose offer superior thermal stability, charge interaction capabilities, and mechanical integrity, which are crucial for maintaining photocatalyst performance during repeated use [29]. In particular, their complementary charge characteristics, cationic (CHT) and neutral/anionic (BC), promote synergistic electrostatic interactions with mixed-metal oxides, leading to improved catalyst dispersion and pollutant adsorption efficiency.

In this study, we report the synthesis of MnCo_2_O_4_ nanoparticles (NPs) via a precipitation-hydrothermal aging method, employing biodegradable stabilizers such as chitosan (CHT) and biomass-derived cellulose (BC) to tailor the nanoparticles’ morphology, surface area, and stability. The effects of these stabilizers on the photocatalytic degradation efficiency of MB under UV-rich solar irradiation were systematically evaluated. This work demonstrates the potential of biopolymer-assisted synthesis to produce sustainable, high-performance photocatalysts for environmentally friendly water remediation.

## 2. Experimental Procedure

### 2.1. Materials

All chemicals used in this study were of analytical grade and utilized without further purification. Manganese(II) chloride tetrahydrate (MnCl_2_·4H_2_O, ≥98%), cobalt(II) chloride hexahydrate (CoCl_2_·6H_2_O, 98%), ammonium hydroxide (NH_4_OH, 30–33% NH_3_ in H_2_O), biomass cellulose (BC, 140–1400 mesh), methylene blue (MB, 100%), and chitosan (CHT, high molecular weight ~310,000–375,000 Da) were all purchased from Sigma-Aldrich (Merck KGaA, St. Louis, MO, USA). Deionized double-distilled water was used throughout all experiments to maintain reproducibility during synthesis and photocatalytic evaluation.

### 2.2. Synthesis of MnCo_2_O_4_ Nanoparticles

The synthesis of MnCo_2_O_4_ nanoparticles (NPs) was conducted through a precipitation-assisted hydrothermal method, incorporating biodegradable stabilizers to tailor nanoparticle morphology and surface characteristics. For the biomass-cellulose (BC)-stabilized MnCo_2_O_4_, 1.6 g of biomass-derived cellulose was dispersed in 100 mL of deionized water in a glass vessel and stirred at room temperature (~25 °C) for 30 min to ensure complete dissolution and homogeneity. Separately, aqueous solutions of MnCl_2_·4H_2_O (0.396 g in 15 mL) and CoCl_2_·6H_2_O (0.795 g in 15 mL) were prepared using deionized water and added dropwise to the BC solution under continuous magnetic stirring at 400 rpm. The mixture was stirred for an additional 10 min to achieve complete complexation between the metal cations and cellulose hydroxyl groups. Subsequently, ammonium hydroxide (NH_4_OH, 25%) was diluted to 20 mL and added dropwise to the reaction mixture to initiate co-precipitation. The pH was carefully adjusted to 9, as this value favors the formation of mixed-metal hydroxides, which serve as precursors to MnCo_2_O_4_. The reaction was maintained under constant stirring for 20 min to ensure uniform nucleation and growth of the precipitate. The resulting suspension was then transferred to a 100 mL Teflon-lined stainless-steel autoclave and subjected to hydrothermal treatment at 180 °C for 6 h. After cooling naturally to room temperature, the solid product was separated by centrifugation at 6000 rpm for 10 min. The obtained precipitate was washed repeatedly with double-distilled water and ethanol until the supernatant reached a neutral pH (~7) to remove any unreacted ions and impurities. Finally, the sample was dried in an oven at 60 °C for 12 h, yielding the MnCo_2_O_4_–BC nanocatalyst. A similar procedure was followed for the synthesis of chitosan-stabilized MnCo_2_O_4_ (MnCo_2_O_4_–CHT), where 1.6 g of chitosan was first dissolved in 100 mL of 1% (*v*/*v*) acetic acid solution to obtain a clear polymeric matrix before the addition of metal precursors. The pH was adjusted to ~9.3 during NH_4_OH addition to ensure proper co-precipitation.

### 2.3. Characterization

The MnCo_2_O_4_ nanoparticles were extensively characterized using a range of advanced analytical instruments. The crystalline structure was examined using an X-ray diffractometer (Maxima-X, Rigaku Corporation, Tokyo, Japan) equipped with Cu Kα radiation (λ = 1.5406 Å) and scanned over a 2θ range of 10°–80°. The surface chemical composition and elemental oxidation states were analyzed by X-ray photoelectron spectroscopy (XPS) using an ESCA 250Xi spectrometer (Thermo Fisher Scientific, Waltham, MA, USA). Fourier transform infrared (FTIR) spectroscopy was performed using a Shimadzu IRTracer-100 spectrometer (Shimadzu Corporation, Kyoto, Japan) to identify surface functional groups and chemical bonding. The specific surface area and pore characteristics were determined via nitrogen adsorption–desorption isotherms at 77 K using a NOVA 4200e surface area analyzer (Quantachrome Instruments, Boynton Beach, FL, USA), with samples pre-activated at 110 °C for 12 h. Morphological analysis was performed using a field-emission scanning electron microscope (FESEM, JSM-7610F, JEOL Ltd., Tokyo, Japan) after sputter-coating the samples with gold. The thermal stability and decomposition behavior were examined by thermogravimetric analysis (TGA) using an STA 449 F3 Jupiter thermal analyzer (NETZSCH-Gerätebau GmbH, Selb, Germany). Finally, optical absorption spectra were recorded using a UV–Vis spectrophotometer (V-670, JASCO Corporation, Tokyo, Japan) over a wavelength range of 200–900 nm. Collectively, these techniques provided comprehensive insights into the structural, morphological, optical, and thermal properties of the synthesized MnCo_2_O_4_ nanospinels.

### 2.4. Photocatalytic Performance

The photocatalytic degradation of MB dye was conducted under UV-rich solar irradiation (AM 1.5 G, 100 mW/cm^2^) to ensure reproducibility and controlled irradiation intensity. Initially, 60 mg of AC@PSS was added to 25 mL of an MB solution at 10 mg/L. The mixture was kept in the dark for 90 min to achieve adsorption equilibrium before being exposed to UV-rich solar light. At regular intervals, 3 mL samples of the reaction mixture were withdrawn, centrifuged to separate the catalyst, and analyzed by UV-Vis spectrophotometry at 660 nm (λmax). The photocatalytic degradation efficiency (%) was calculated using Equation (1).

(1) $$photocatalytic\;degradation\left(\%\right)=\frac{C_{o}-C_{t}}{C_{o}}\times 100$$

Here, C_o_ and C_t_ represent the initial concentration and the concentration at time t after degradation.

## 3. Result and Discussion

### 3.1. Characterization of the Fabricated MnCo_2_O_4_ Samples

**Figure 1:**
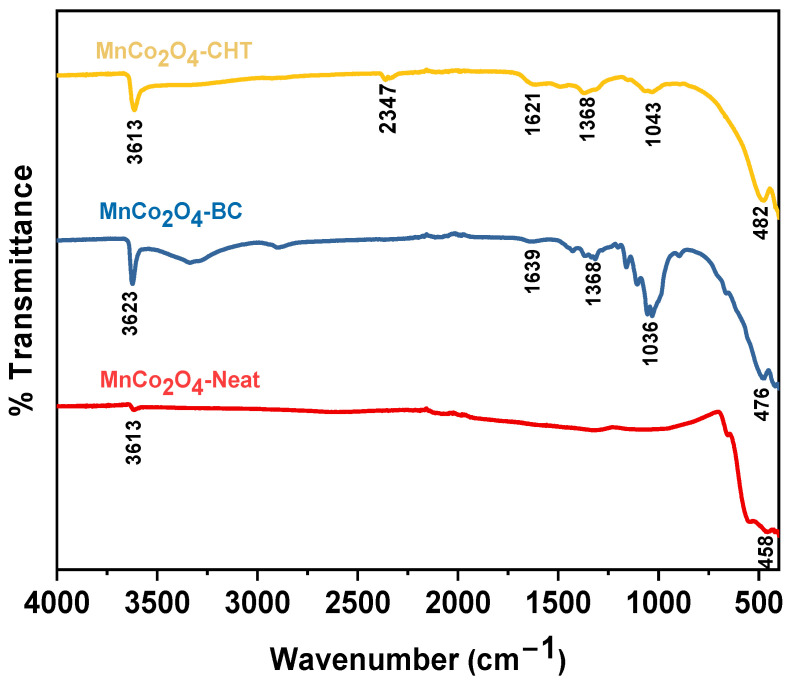
FTIR spectra of MnCo_2_O_4_-Neat, MnCo_2_O_4_-CHT, and MnCo_2_O_4_-BC synthesized using various biodegradable stabilizers.

Figure 1 illustrates the FTIR spectra of MnCo_2_O_4_ synthesized with no stabilizer (MnCo_2_O_4_-Neat), with biomass-cellulose (MnCo_2_O_4_-BC), and with chitosan (MnCo_2_O_4_-CHT) as biodegradable stabilizers. The analysis highlights key functional groups, structural features, and the influence of stabilizers on the MnCo_2_O_4_ nanoparticles, emphasizing the role of surface modifications in enhancing their catalytic properties. All three samples exhibit broad absorption bands in the range of 3613–3623 cm^−1^, attributed to the O–H stretching vibrations of adsorbed water molecules or surface hydroxyl groups. These hydrophilic groups are crucial for catalytic processes as they enhance interaction with polar dye molecules like methylene blue (MB). The distinct peaks observed at 458–482 cm^−1^ and 1036–1043 cm^−1^ correspond to Co–O and Mn–O stretching vibrations, respectively, confirming the successful formation of the MnCo_2_O_4_ spinel structure across all samples. The MnCo_2_O_4_-Neat sample, synthesized without any stabilizer, shows relatively weaker and less pronounced peaks compared to the stabilized samples. This indicates a simpler surface chemistry with fewer functional groups, which might limit its interaction with reactants and potentially reduce its catalytic efficiency. In contrast, MnCo_2_O_4_-BC, synthesized using biomass-cellulose, displays notable peaks at 1639 cm^−1^ and 1368 cm^−1^, corresponding to C=O and C–O stretching vibrations. These peaks signify the presence of cellulose residues on the nanoparticle surface, which likely enhance its adsorption capabilities by providing additional polar functional groups. This surface modification may improve the photocatalytic degradation of MB by increasing the interaction between the dye and the catalyst surface. Similarly, MnCo_2_O_4_-CHT, synthesized with chitosan as the stabilizer, exhibits strong peaks at 1621 cm^−1^ and 1368 cm^−1^, corresponding to amide I (C=O stretching) and amide III (C–N stretching and N–H bending) vibrations. The presence of these functional groups suggests a robust interaction between chitosan and the MnCo_2_O_4_ nanoparticles, leading to a modified surface with enhanced adsorption sites. The sharper peaks indicate a stronger impact of chitosan in tailoring the surface chemistry compared to cellulose, which may contribute to improved dispersion and reactivity in photocatalytic applications. The comparison across the three samples highlights the critical role of stabilizers in modifying the surface properties of MnCo_2_O_4_ nanoparticles. While MnCo_2_O_4_-Neat has a simpler structure, the incorporation of biomass-cellulose and chitosan introduces additional functional groups, enhancing the nanoparticles’ ability to adsorb and degrade MB. These modifications are expected to improve the photocatalytic activity of MnCo_2_O_4_ by facilitating better interaction with MB and enhancing the degradation process. In conclusion, the FTIR spectra confirm the successful synthesis of MnCo_2_O_4_ spinel nanoparticles and underline the significant influence of biodegradable stabilizers on their surface chemistry [30,31,32,33]. The results demonstrate that the choice of stabilizer, whether biomass-cellulose or chitosan, plays a crucial role in tailoring the nanoparticles’ properties for environmental remediation applications, such as the photocatalytic degradation of MB. This work underscores the potential of bio-degradable stabilizers to enhance the performance of MnCo_2_O_4_ in catalytic processes, paving the way for more efficient and sustainable solutions in water treatment.

**Figure 2:**
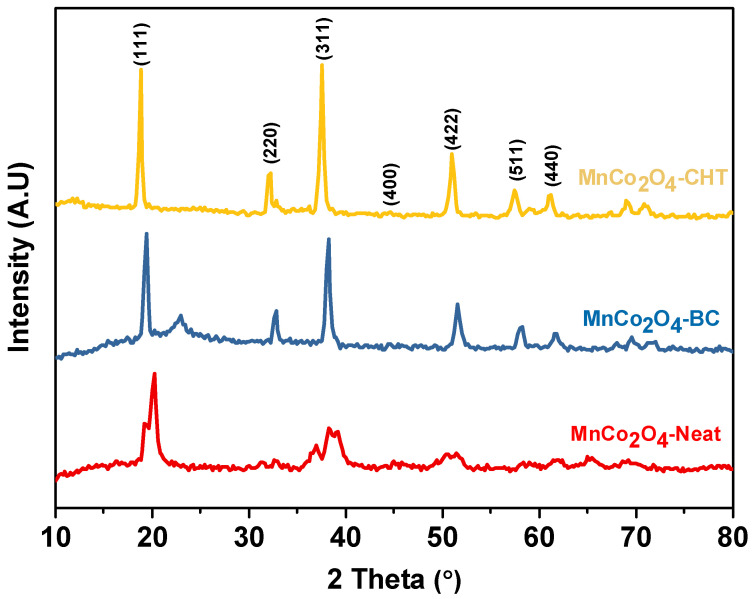
XRD patterns of MnCo_2_O_4_-Neat, MnCo_2_O_4_-CHT, and MnCo_2_O_4_-BC synthesized using various biodegradable stabilizers.

Figure 2 displays the X-ray diffraction (XRD) patterns of MnCo_2_O_4_ synthesized with and without stabilizers: MnCo_2_O_4_-Neat (without a stabilizer), MnCo_2_O_4_-BC (using biomass-cellulose as a stabilizer), and MnCo_2_O_4_-CHT (using chitosan as a stabilizer). The diffraction peaks at the 2θ values of 18.71, 32.11, 37.6, 44.4, 50.9, 57.56, and 61.2°, which are correspond to the characteristic planes of the MnCo_2_O_4_ spinel structure, including (111), (220), (311), (400), (511), and (440). These patterns confirm the successful formation of the spinel MnCo_2_O_4_ crystalline phase while demonstrating the significant impact of the stabilizers on the material’s crystallinity and structural integrity. The XRD patterns of MnCo_2_O_4_ closely align with the standard JCPDS card no. 023-1237, confirming the successful formation of the expected spinel structure. However, notable differences in the sharpness, intensity, and width of the peaks highlight the influence of stabilizers on the crystallinity and grain size of the nanoparticles. The absence of any additional peaks indicates the phase purity of the synthesized MnCo_2_O_4_ compound. The XRD pattern of MnCo_2_O_4_-Neat shows broader peaks with relatively low intensity, indicating lower crystallinity and smaller grain size. The absence of a stabilizing agent likely led to uncontrolled nucleation and growth, resulting in less uniform particles and higher structural disorder. These characteristics could negatively impact the material’s performance, as lower crystallinity may hinder charge carrier mobility in photocatalytic applications. The XRD pattern of MnCo_2_O_4_-BC reveals sharper and more intense peaks compared to MnCo_2_O_4_-Neat, reflecting improved crystallinity. Biomass-cellulose acts as a stabilizing scaffold during synthesis, facilitating controlled nucleation and growth of the crystalline phase. The cellulose matrix likely ensures a more uniform dispersion of the metal precursors, preventing agglomeration and promoting the formation of well-ordered particles. This enhancement in crystallinity contributes to improved material properties, such as better structural stability and potential photocatalytic activity. Additionally, the MnCo_2_O_4_-CHT sample exhibits the sharpest and most intense diffraction peaks, indicating the highest degree of crystallinity among the three samples. Chitosan’s chelating ability, attributed to its functional groups (e.g., –NH_2_ and –OH), plays a critical role in coordinating metal ions during synthesis. This interaction promotes a highly ordered arrangement of atoms in the spinel structure. Additionally, the polymeric network of chitosan provides a template that ensures uniform nucleation and growth, resulting in enhanced structural properties. The degree of crystallinity has direct implications for the photocatalytic performance of MnCo_2_O_4_. Higher crystallinity, as observed in MnCo_2_O_4_-BC and MnCo_2_O_4_-CHT, reduces defect sites and improves charge carrier mobility, essential for efficient photocatalysis. Additionally, the functional groups introduced by the stabilizers, such as hydroxyl and amino groups, enhance surface adsorption of methylene blue, further improving the degradation efficiency. The superior crystallinity and surface properties of MnCo_2_O_4_-CHT suggest that it may exhibit the best photocatalytic performance among the three samples. The XRD analysis underscores the critical role of stabilizers in influencing the structural properties of MnCo_2_O_4_ nanoparticles. While the MnCo_2_O_4_-Neat sample demonstrates lower crystallinity, the use of biomass-cellulose and chitosan significantly enhances the crystallinity and structural order. Among the stabilizers, chitosan proves to be the most effective, yielding the sharpest peaks and the highest crystallinity. These structural improvements, facilitated by the stabilizers, are expected to enhance the photocatalytic degradation efficiency of methylene blue, demonstrating the importance of stabilizer selection in optimizing material properties for environmental remediation applications. The crystallite size (t) was estimated using Scherer’s formula [33]:

(2) $$t=\frac{0.9\lambda }{\beta cos\theta }$$

where, t is the average crystallites size, λ is the wavelength of the X-ray (Cu Kα1, 1.5418 Å), β is the full-width half maximum, and θ is the diffraction angle. The calculated crystallite sizes are 19.1 nm for MnCo_2_O_4_-Neat, 16.4 nm for MnCo_2_O_4_-BC, and 13.3 nm for MnCo_2_O_4_-CHT. The differences in crystallite size are attributed to the influence of the specific additives used during the synthesis of each sample. The average lattice parameter (*a*) for all samples was calculated using the equation *a* = d$\surd (h^{2}+k^{2}+l^{2})$, based on the (311) plane. The obtained lattice parameters were 8.463 Å for MnCo_2_O_4_-Neat, 8.475 Å for MnCo_2_O_4_-BC, and 8.482 Å for MnCo_2_O_4_-CHT, all of which are close to the theoretical value of 8.49 Å [34,35].

**Figure 3:**
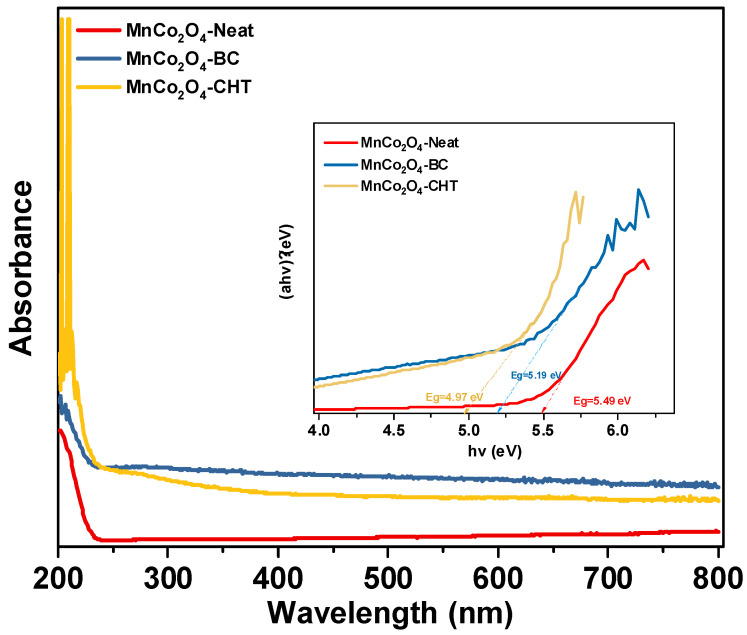
UV–Vis absorption spectra and the corresponding Tauc plots (inset) of MnCo_2_O_4_-Neat, MnCo_2_O_4_-CHT, and MnCo_2_O_4_-BC synthesized using various biodegradable stabilizers.

The UV–Vis absorption spectra and corresponding Tauc plots (inset) (Figure 3) of MnCo_2_O_4_-Neat, MnCo_2_O_4_-CHT, and MnCo_2_O_4_-BC provide valuable insights into the optical properties of these materials and the effect of biodegradable stabilizers on their electronic structure. All samples exhibit significant absorption in the UV and visible regions, indicative of their ability to absorb light across a broad wavelength range, which is beneficial for photocatalytic applications. The absorption onset in the visible region corresponds to the charge transfer transitions between the Co^3+^/Mn^3+^ d-orbitals and the O^2−^ p-orbitals, a characteristic of the MnCo_2_O_4_ spinel structure. The MnCo_2_O_4_-Neat sample shows a lower absorption intensity in the visible region compared to MnCo_2_O_4_-BC and MnCo_2_O_4_-CHT, indicating that the absence of stabilizers results in less efficient light-harvesting capability. The MnCo_2_O_4_-BC sample, synthesized with biomass-cellulose, demonstrates enhanced light absorption in the visible region, likely due to the improved crystallinity and better dispersion of particles facilitated by the stabilizer. MnCo_2_O_4_-CHT, synthesized using chitosan as a stabilizer, exhibits the highest absorption intensity, which can be attributed to the strong interaction between chitosan’s functional groups and the metal ions during synthesis, leading to improved particle homogeneity and surface modification. The Tauc plots (**inset**) were used to estimate the optical band gaps of the samples. The calculated band gap energies are approximately 5.49 eV, 5.19 V, and 4.97 eV for MnCo_2_O_4_-Neat, MnCo_2_O_4_-BC, and MnCo_2_O_4_-CHT, respectively. The slight reduction in the band gap for MnCo_2_O_4_-BC and MnCo_2_O_4_-CHT compared to MnCo_2_O_4_-Neat suggests that the stabilizers introduce surface states or modify the electronic structure, enhancing visible light absorption. Chitosan, in particular, appears to lower the band gap the most, potentially due to its chelating effects and the incorporation of functional groups that alter the band structure. The results demonstrate that the choice of stabilizer significantly affects the optical properties of MnCo_2_O_4_. The improved light absorption and reduced band gap observed in MnCo_2_O_4_-BC and MnCo_2_O_4_-CHT highlight the importance of biodegradable stabilizers in tailoring the material’s electronic and optical properties, making them more suitable for photocatalytic applications such as the degradation of methylene blue. These findings underscore the critical role of stabilizer selection in optimizing MnCo_2_O_4_ for enhanced photocatalytic performance [30,32,34,36].

**Figure 4:**
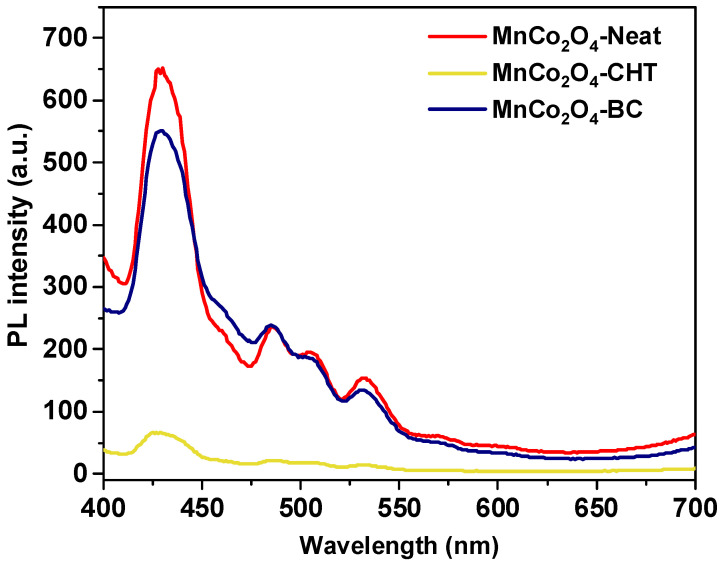
PL spectra of the developed MnCo_2_O_4_-Neat, MnCo_2_O_4_-CHT, and MnCo_2_O_4_-BC synthesized using various biodegradable stabilizer.

The photoluminescence (PL) spectra in Figure 4 illustrate the recombination rates of photogenerated electron-hole pairs for MnCo_2_O_4_-Neat, MnCo_2_O_4_-BC, and MnCo_2_O_4_-CHT, synthesized with and without biodegradable stabilizers. PL spectra are a critical tool to evaluate charge recombination processes in photocatalysts, as lower PL intensity indicates more effective separation of charge carriers, enhancing photocatalytic performance. The MnCo_2_O_4_-Neat sample displays the highest PL intensity, indicating a higher recombination rate of electron-hole pairs. The absence of stabilizers results in minimal surface modifications or structural optimization, contributing to inefficient charge separation and limiting photocatalytic efficiency. For MnCo_2_O_4_-BC, synthesized with biomass-cellulose, the PL intensity is significantly reduced compared to MnCo_2_O_4_-Neat. This reduction can be attributed to the stabilizing effect of cellulose, which enhances the crystalline quality and introduces surface modifications. These changes lower the recombination rate of charge carriers, improving photocatalytic efficiency. On the other hand, MnCo_2_O_4_-CHT, synthesized using chitosan, exhibits the lowest PL intensity among the samples, suggesting the most effective suppression of charge recombination. The strong chelating and templating effects of chitosan during synthesis likely improve structural homogeneity and introduce functional groups that act as charge trapping sites, further enhancing charge separation and transport. The observed differences in PL intensity correlate directly with the electronic properties of the materials. The HOMO (highest occupied molecular orbital) and LUMO (lowest unoccupied molecular orbital) levels of MnCo_2_O_4_ are critical for charge transfer processes. The incorporation of biodegradable stabilizers appears to influence these levels: (i): A higher recombination rate of MnCo_2_O_4_-Neat, suggests less effective overlap or mismatch between HOMO and LUMO, leading to rapid electron-hole recombination. (ii) The cellulose stabilizer of MnCo_2_O_4_-BC improves HOMO-LUMO alignment by modifying the surface energy states, facilitating better charge transfer. (iii) Chitosan introduces functional groups that lower the recombination rate by optimizing HOMO-LUMO interactions of MnCo_2_O_4_-CHT and creating effective pathways for charge separation. The results of this study are supported by previous research highlighting the role of surface modifications and stabilizers in enhancing charge separation and suppressing recombination in photocatalysts. For example, the Ag_2_CO_3_/AgX heterojunctions synthesized by Dong et al. (2014) demonstrated improved photocatalytic activity due to efficient charge carrier separation and suppressed recombination at the interface, which aligns with the reduced PL intensity observed for MnCo_2_O_4_-BC and MnCo_2_O_4_-CHT [37]. Similarly, Yadav et al. (2017) reported that tungsten-doped TiO_2_/rGO composites exhibited reduced PL intensity due to the presence of tungsten as an electron trap, a phenomenon comparable to the charge trapping and improved HOMO-LUMO interactions introduced by chitosan in MnCo_2_O_4_-CHT [38]. Moreover, Fakhravar et al. (2020) showed that the incorporation of Cu_2_S and Ag_2_S in a ternary heterostructure significantly enhanced charge carrier mobility and reduced recombination, similar to the role of biomass-cellulose and chitosan in optimizing MnCo_2_O_4_’s surface and electronic properties [39]. Finally, Du et al. (2021) [40,41] highlighted the role of functional groups in reducing charge recombination in a g-C_3_N_4_/MnO_2_/GO heterojunction, corroborating our findings that chitosan enhances surface functionality and reduces recombination in MnCo_2_O_4_-CHT. These studies collectively support our results, emphasizing the effectiveness of biodegradable stabilizers in improving the photocatalytic performance of MnCo_2_O_4_.

**Figure 5:**
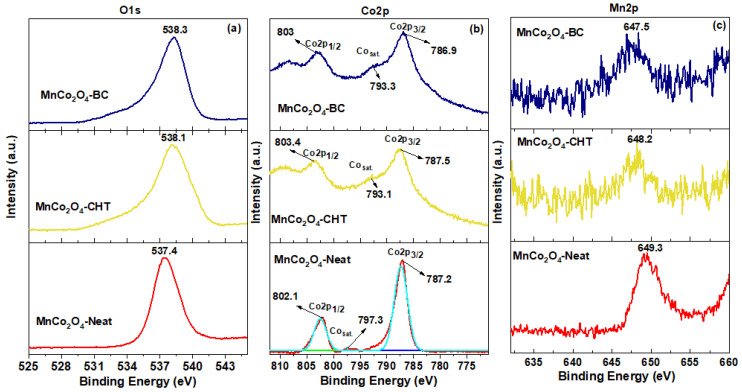
XPS deconvoluted spectra (**a**) O1s, (**b**) Co2p, and (**c**) Mn2p of the developed MnCo_2_O_4_-Neat, MnCo_2_O_4_-CHT, and MnCo_2_O_4_-BC synthesized using various biodegradable stabilizer.

The X-ray photoelectron spectroscopy (XPS) results provide a comprehensive understanding of the surface chemistry and electronic structure of MnCo_2_O_4_-Neat, MnCo_2_O_4_-BC, and MnCo_2_O_4_-CHT, shedding light on the factors driving their photocatalytic performance (Figure 5). The analysis of the O1s, Co2p, and Mn2p spectra highlights the critical role of biodegradable stabilizers in modifying the surface properties of MnCo_2_O_4_ and correlates directly with their observed photocatalytic efficiency. The O1s spectra reveal the presence of surface-adsorbed oxygen species, such as hydroxyl groups and chemisorbed water, which are crucial for generating reactive oxygen species (ROS) during photocatalysis. The higher binding energy observed for MnCo_2_O_4_-CHT and MnCo_2_O_4_-BC suggests an enhanced abundance of these active oxygen species, likely due to the interaction between the stabilizers and the MnCo_2_O_4_ framework. This increased reactivity facilitates the adsorption and activation of methylene blue (MB) molecules, contributing to the superior degradation performance observed for MnCo_2_O_4_-CHT (96% MB degradation) and MnCo_2_O_4_-BC (65%), compared to MnCo_2_O_4_-Neat (45%). The Co2p spectra further emphasize the impact of stabilizers on the electronic structure. The increased Co^3+^/Co^2+^ ratio in MnCo_2_O_4_-CHT reflects enhanced redox activity, which is critical for efficient charge transfer and reduced electron-hole recombination. Chitosan’s chelating effect likely stabilizes Co^3+^ species, while the templating role of cellulose introduces structural modifications that similarly improve the Co^3+^ contribution in MnCo_2_O_4_-BC. These redox-active sites promote photocatalytic reactions by facilitating the transfer of electrons to oxygen molecules, generating ROS to degrade MB. Similarly, the Mn2p spectra highlight the stabilization of Mn^3+^ and Mn^4+^ states, particularly in MnCo_2_O_4_-CHT and MnCo_2_O_4_-BC. The highest binding energy shift in MnCo_2_O_4_-CHT reflects the presence of a higher proportion of Mn^4+^ species, which are critical for enhancing charge carrier dynamics and oxidative degradation. The presence of Mn^4+^ improves electron-hole separation, reducing recombination rates and further contributing to the high catalytic efficiency of MnCo_2_O_4_-CHT. In contrast, MnCo_2_O_4_-Neat shows lower Mn^4+^ contributions, consistent with its lower photocatalytic performance. These XPS findings align closely with the photocatalytic activity of the samples. The superior performance of MnCo_2_O_4_-CHT is attributed to its optimized surface chemistry, with an abundance of reactive oxygen species, enhanced Co^3+^/Co^2+^ ratios, and stabilized Mn^4+^ states. MnCo_2_O_4_-BC also exhibits improved activity due to the templating role of cellulose, which enhances surface reactivity. In contrast, the lower activity of MnCo_2_O_4_-Neat reflects its less favorable surface and electronic properties. In conclusion, the XPS analysis establishes a strong correlation between the surface chemistry of MnCo_2_O_4_ nanospinels and their photocatalytic efficiency. The use of chitosan and cellulose as stabilizers significantly enhances the catalytic activity by improving the availability of active oxygen species, tuning redox states, and optimizing charge transfer. These findings underline the critical role of biodegradable stabilizers in designing efficient and sustainable photocatalysts for environmental remediation.

**Figure 6:**
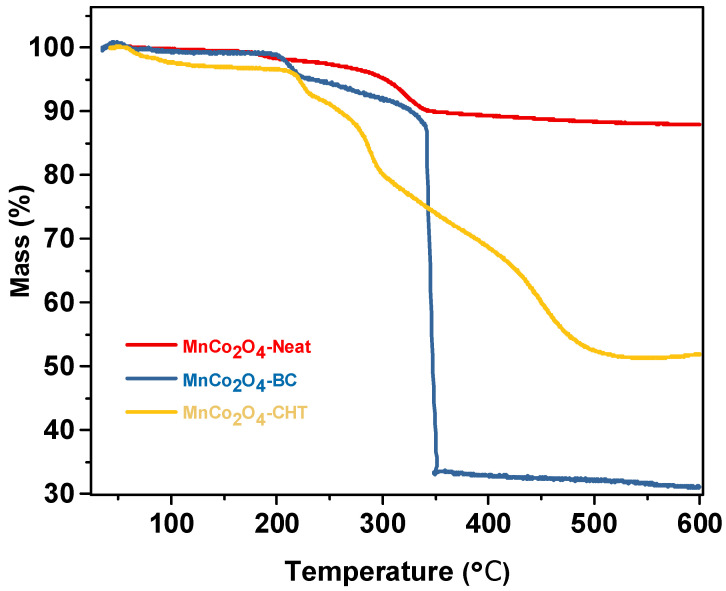
TGA profiles of MnCo_2_O_4_-Neat, MnCo_2_O_4_-CHT, and MnCo_2_O_4_-BC synthesized using various biodegradable stabilizers.

Figure 6 presents the thermogravimetric analysis (TGA) profiles of MnCo_2_O_4_-Neat, MnCo_2_O_4_-CHT, and MnCo_2_O_4_-BC, illustrating their thermal stability and the effect of biodegradable stabilizers on the decomposition behavior of the synthesized materials. The profiles highlight the weight loss percentages as a function of temperature, indicating distinct thermal degradation steps for each sample. The TGA curve for MnCo_2_O_4_-Neat shows minimal weight loss up to ~200 °C, which can be attributed to the removal of physically adsorbed water and surface-bound moisture. The absence of significant weight loss in this range reflects the lack of organic additives or stabilizers in this sample. Beyond 200 °C, the material exhibits only slight decomposition, indicating good thermal stability, with no major thermal events up to 600 °C. This behavior demonstrates that MnCo_2_O_4_-Neat retains its structural integrity well at high temperatures. The MnCo_2_O_4_-BC sample shows a two-step weight loss process. The initial weight loss below 200 °C corresponds to the evaporation of physically adsorbed water and the decomposition of small organic residues introduced by biomass-cellulose. The second and more significant weight loss occurs between 200 °C and 400 °C, which is attributed to the thermal decomposition of cellulose and its derivatives. This substantial weight loss reflects the presence of organic components from the biomass-cellulose stabilizer. Above 400 °C, the decomposition stabilizes, indicating the thermal resilience of the inorganic MnCo_2_O_4_ framework after the organic components are removed [41]. The TGA curve for MnCo_2_O_4_-CHT exhibits a similar two-step degradation profile. The initial weight loss below 200 °C is attributed to the removal of adsorbed water and volatile components. The more pronounced weight loss between 200 °C and 400 °C corresponds to the decomposition of chitosan, including its amine (-NH_2_) and hydroxyl (-OH) functional groups. The slower degradation above 400 °C compared to MnCo_2_O_4_-BC indicates that the chitosan stabilizer contributes to improved thermal stability of the material [42]. The presence of biodegradable stabilizers (biomass-cellulose and chitosan) significantly affects the thermal degradation profile of MnCo_2_O_4_. Both MnCo_2_O_4_-BC and MnCo_2_O_4_-CHT exhibit higher overall weight loss compared to MnCo_2_O_4_-Neat due to the decomposition of organic components in the stabilizers. Chitosan, however, appears to enhance thermal stability beyond 400 °C compared to cellulose, potentially due to its stronger interaction with the MnCo_2_O_4_ framework through chelation and surface modifications. In conclusion, chitosan provides superior stabilization compared to biomass-cellulose, resulting in higher thermal resilience. These findings underline the importance of stabilizers in tailoring the thermal and functional properties of MnCo_2_O_4_ for advanced catalytic applications.

**Figure 7:**
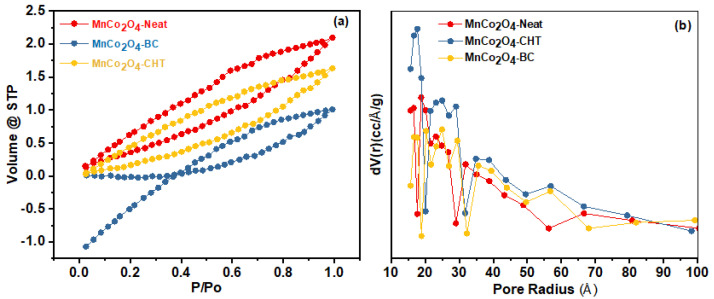
(**a**) N2 adsorption–desorption isotherm, and (**b**) pore size distribution of MnCo_2_O_4_-Neat, MnCo_2_O_4_-CHT, and MnCo_2_O_4_-BC synthesized using various biodegradable stabilizers.

The nitrogen adsorption–desorption isotherms and pore size distribution of MnCo_2_O_4_-Neat, MnCo_2_O_4_-CHT, and MnCo_2_O_4_-BC (Figure 7) reveal significant differences in surface area and pore characteristics, as summarized in Table 1. These differences are attributed to the influence of biodegradable stabilizers—chitosan (CHT) and biomass-cellulose (BC)—used during the synthesis process. The analysis provides critical insights into the role of stabilizers in tailoring the textural properties of MnCo_2_O_4_ nanospinels for optimized catalytic performance. The isotherms in Figure 7a exhibit a type IV profile with an H3 hysteresis loop for all samples, indicative of mesoporous materials. The MnCo_2_O_4_-Neat sample, synthesized without any stabilizer, demonstrates the greatest BET surface area (33.44 m^2^/g) and a smaller pore size (26.2 nm) compared to the stabilized samples. This result suggests that the absence of stabilizers enables higher surface exposure during synthesis, resulting in smaller pores and a higher surface area. However, the limited structural control may lead to non-uniformity and less organized porosity, as observed in the broader pore distribution in Figure 7b. For MnCo_2_O_4_-CHT, the surface area (27.42 m^2^/g) is reduced compared to MnCo_2_O_4_-Neat, but the average pore diameter increases to 29.1 nm. The role of chitosan as a stabilizer is evident in the more uniform pore size distribution and increased pore volume (0.0019 cm^3^/g). The chelating ability of chitosan likely facilitates the formation of larger, more organized pores by controlling the aggregation of particles during synthesis. This enhanced structural organization may improve accessibility for catalytic processes, as demonstrated in other studies where chitosan templates increased the porosity and uniformity of nanostructured materials [43]. Similarly, MnCo_2_O_4_-BC, synthesized with biomass-cellulose as a stabilizer, exhibits a slightly lower surface area (29.32 m^2^/g) than MnCo_2_O_4_-Neat but shows an increase in pore diameter (27.3 nm) and reduced pore volume (0.0013 cm^3^/g). Biomass-cellulose acts as a template to control the particle aggregation and promotes the formation of larger, more accessible pores. The stabilizer’s role in modifying pore size is supported by similar findings in cellulose-stabilized metal oxides, where enhanced porosity was attributed to the templating action of cellulose fibers during the sol-gel process [41]. The pore size distributions in Figure 7b further confirm that both chitosan and cellulose stabilize the MnCo_2_O_4_ nanostructures by preventing excessive pore collapse and aggregation, resulting in larger and more uniform pores compared to MnCo_2_O_4_-Neat. This structural modification aligns with research on the role of stabilizers in tailoring mesoporous materials for improved adsorption and catalytic properties [44]. In summary, the use of chitosan and biomass-cellulose as stabilizers introduces significant structural differences in the MnCo_2_O_4_ samples. While MnCo_2_O_4_-Neat exhibits the highest surface area, the stabilized samples show more uniform pore structures and larger pore sizes, which may enhance their catalytic efficiency. These findings emphasize the importance of biodegradable stabilizers in optimizing the textural properties of MnCo_2_O_4_ for applications such as photocatalytic degradation and environmental remediation.

**Figure 8:**
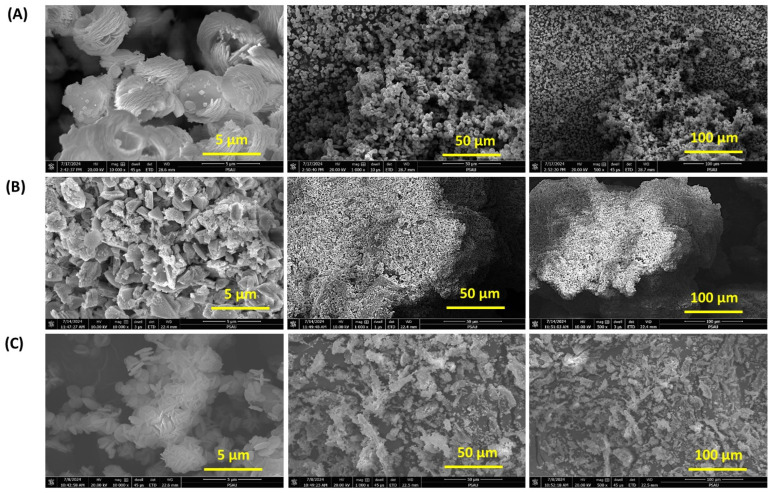
FESEM images at different magnifications of (**A**) MnCo_2_O_4_-Neat, (**B**) MnCo_2_O_4_-CHT, and (**C**) MnCo_2_O_4_-BC nanospinel.

The field emission scanning electron microscopy (FESEM) images in Figure 8 provide detailed insights into the surface morphology and microstructure of MnCo_2_O_4_ synthesized with and without biodegradable stabilizers: MnCo_2_O_4_-Neat, MnCo_2_O_4_-CHT, and MnCo_2_O_4_-BC. The variations in morphology among the samples highlight the significant impact of stabilizers—chitosan (CHT) and biomass-cellulose (BC)—on the nucleation and growth of MnCo_2_O_4_ nanostructures. The FESEM images of MnCo_2_O_4_-Neat (Figure 8A) reveal a relatively disordered and aggregated structure with non-uniform particle shapes and sizes. This irregular morphology can be attributed to the absence of stabilizers during synthesis, leading to uncontrolled nucleation and growth. The aggregated particles lack well-defined boundaries, which may negatively impact the surface area and catalytic activity. This observation aligns with previous studies showing that stabilizer-free synthesis often results in non-homogeneous particle distribution due to the lack of templating or chelating agents. In contrast, MnCo_2_O_4_-CHT (Figure 8B) demonstrates a more organized structure with smaller, uniformly distributed particles. The chitosan stabilizer facilitates better control over particle size and distribution due to its chelating properties and ability to interact with metal ions during synthesis. The observed morphology suggests that chitosan acts as a template, preventing excessive particle aggregation and promoting the formation of nanostructures with enhanced surface area and porosity. These findings are consistent with previous reports where chitosan was shown to improve the morphology and dispersion of metal oxide nanoparticles for catalytic applications [43]. The morphology of MnCo_2_O_4_-BC (Figure 8C) reveals a fibrous and interconnected structure, characteristic of the templating effect of biomass-cellulose. The cellulose fibers provide a framework for the nucleation and growth of MnCo_2_O_4_, resulting in a more porous structure with larger surface areas. The observed morphology is consistent with the role of cellulose as a structural template, as reported in similar studies where cellulose-derived frameworks enhanced the structural integrity and porosity of metal oxides [41]. The fibrous morphology may facilitate better mass transfer and adsorption of target molecules, which is critical for photocatalytic applications. Comparing the three samples, it is evident that the use of biodegradable stabilizers significantly influences the morphology of MnCo_2_O_4_. The more defined and porous structures observed in MnCo_2_O_4_-CHT and MnCo_2_O_4_-BC are likely to enhance their catalytic performance by increasing the available surface area and improving the accessibility of active sites. The stabilizer-induced morphological control highlights the importance of employing templating agents to tailor the structural properties of nanospinels for specific applications. In conclusion, the FESEM analysis demonstrates that the absence of stabilizers in MnCo_2_O_4_-Neat leads to disordered aggregation, whereas the use of chitosan and cellulose results in more uniform and organized structures. Chitosan yields smaller, well-dispersed nanoparticles, while cellulose produces a fibrous, interconnected network. These findings underscore the role of stabilizers in enhancing the structural and functional properties of MnCo_2_O_4_, making them more suitable for catalytic and environmental applications.

### 3.2. Photocatalytic Performance of the Prepared Nanospinel MnCo_2_O_4_

**Figure 9:**
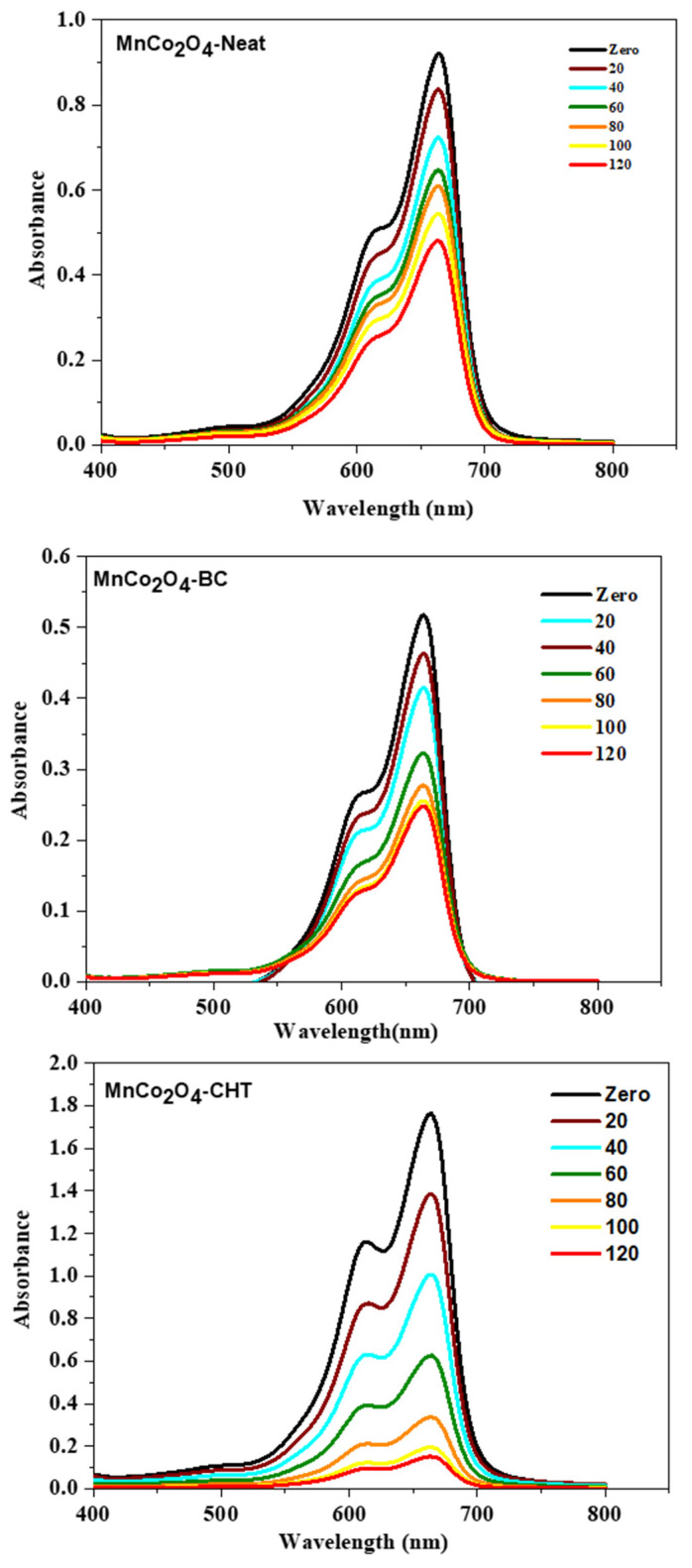
Time-dependent variations in absorption spectrum of MB during under UV-rich solar irradiation photocatalytic degradation in presence MnCo_2_O_4_-Neat, MnCo_2_O_4_-BC, and MnCo_2_O_4_-CHT nanospinel at pH (7).

Spinel oxides, with the general formula AB_2_O_4_, are characterized by their cubic close-packed oxygen lattice and mixed-metal cation distribution between tetrahedral (A-site) and octahedral (B-site) sites. This unique structure facilitates efficient charge transport and separation, which are critical for enhancing photocatalytic activity. Furthermore, spinel oxides exhibit tunable band gaps, high chemical stability, and strong light absorption, making them particularly effective under visible and UV irradiation. These properties can be further enhanced through doping, structural engineering, and surface modifications. This study focuses on the photocatalytic degradation of MB using spinel-structured MnCo_2_O_4_ synthesized with and without biodegradable stabilizers. By examining the effects of structural modifications and stabilizer-assisted synthesis on photocatalytic performance, this work aims to provide insights into the design of advanced spinel-based photocatalysts for wastewater treatment applications. Figure 9 illustrates the time-dependent variations in the absorption spectrum of methylene blue (MB) during photocatalytic degradation under UV-rich solar irradiation, using MnCo_2_O_4_-Neat, MnCo_2_O_4_-BC, and MnCo_2_O_4_-CHT nanospinels at neutral pH (7). The progressive reduction in the intensity of the characteristic MB absorption peak at ~660 nm over 120 min confirms the photocatalysts’ degradation of MB. The comparative performance of the three catalysts highlights the significant influence of stabilizers on the photocatalytic efficiency. For MnCo_2_O_4_-Neat, the absorption spectra exhibit a gradual decrease in MB intensity, achieving 45% degradation at the end of 120 min. The relatively low photocatalytic efficiency can be attributed to the absence of stabilizers, which limits structural control and the availability of active sites on the catalyst surface. This result aligns with earlier findings where unmodified spinel oxides demonstrated moderate photocatalytic activity due to higher recombination rates of photogenerated charge carriers [37]. MnCo_2_O_4_-BC, synthesized with biomass-cellulose as a stabilizer, shows a marked improvement in MB degradation, achieving 65% degradation by the end of 120 min. The enhanced performance can be attributed to the templating effect of cellulose, which improves particle dispersion and increases porosity. These structural improvements facilitate greater light absorption and enhanced interaction with MB molecules, thereby improving photocatalytic activity. Previous studies have demonstrated that cellulose-based stabilizers significantly enhance the structural properties of spinel oxides, enabling higher catalytic performance [41]. The MnCo_2_O_4_–CHT catalyst exhibited the highest degradation efficiency, achieving 96% methylene blue (MB) removal within 120 min. This enhanced performance can be attributed to the synergistic interaction between chitosan and the MnCo_2_O_4_ spinel matrix, which significantly improves both the structural and electronic properties of the catalyst. Chitosan contains abundant amino (–NH_2_) and hydroxyl (–OH) functional groups, which serve multiple roles during synthesis and catalysis. These groups coordinate strongly with Mn^2+^ and Co^2+^ ions, acting as chelating sites that promote uniform nucleation and prevent particle agglomeration, resulting in smaller, well-dispersed nanoparticles with higher active surface areas. Furthermore, the –NH_2_ and –OH groups provide electron-donating capabilities that facilitate charge transfer between the chitosan matrix and the MnCo_2_O_4_ surface, thereby accelerating the separation of photogenerated electron–hole pairs and suppressing recombination losses. This improved charge mobility enhances the generation of reactive oxygen species (ROS), such as •OH and •O_2_^−^ radicals, which drive efficient dye degradation. Additionally, the chitosan backbone serves as a templating framework, stabilizing the spinel structure and optimizing the electronic band alignment for efficient light absorption. These synergistic effects collectively explain the superior photocatalytic activity of MnCo_2_O_4_–CHT, consistent with previous findings that demonstrate the role of chitosan functionalization in improving charge separation and enhancing photocatalytic efficiency in metal oxide nanocomposites [43,44]. The comparative results of the three catalysts underscore the critical role of stabilizers in enhancing the photocatalytic properties of MnCo_2_O_4_ nanospinels. The progressive improvement in performance from MnCo_2_O_4_-Neat to MnCo_2_O_4_-BC and MnCo_2_O_4_-CHT highlights the importance of the structural and electronic modifications imparted by the stabilizers. MnCo_2_O_4_-CHT, in particular, demonstrates exceptional efficiency due to its enhanced charge carrier dynamics and optimized morphology, making it a promising candidate for practical photocatalytic applications. These findings reinforce the potential of using biodegradable stabilizers to tailor the properties of spinel oxides for environmental remediation.

**Figure 10:**
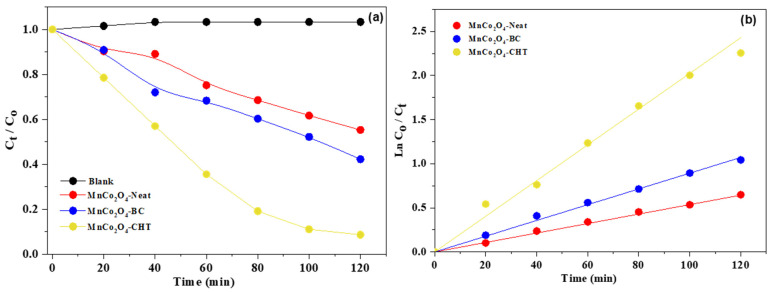
(**a**) Photocatalytic performance (C/C_o_ versus time) and (**b**) Kinetic study (lnC/C_o_ versus time) at pH of 7.

Figure 10 and the accompanying kinetic parameters (Table 2) provide a comprehensive analysis of the photocatalytic degradation of methylene blue (MB) under UV-rich solar irradiation at pH 7 using MnCo_2_O_4_-Neat, MnCo_2_O_4_-BC, and MnCo_2_O_4_-CHT nanospinels. The data illustrate the degradation efficiency (C_t_/C_o_ vs. time), the kinetic behavior (ln(C_o_/C_t_) vs. time), and the apparent rate constants (k_app_) for the three photocatalysts. These results highlight the influence of biodegradable stabilizers on the catalytic performance of MnCo_2_O_4_ and the underlying kinetics of MB degradation. The plot of C_t_/C_o_ vs. time (Figure 10a) demonstrates that MnCo_2_O_4_-CHT achieves the highest degradation efficiency, removing 96% of MB within 120 min. In comparison, MnCo_2_O_4_-BC degrades 65% of MB, while MnCo_2_O_4_-Neat achieves 45% degradation in the same time frame. These trends are further supported by the kinetic study (ln(C_o_/C_t_) vs. time vs., Figure 10b), which shows a linear relationship for all samples, confirming that the degradation process follows pseudo-first-order kinetics. The kinetic parameters in the table reinforce these observations. MnCo_2_O_4_-CHT exhibits the highest apparent rate constant (k_app_ = 0.0203 min^−1^) and the shortest half-life (t_1/2_ = 34.2 min), indicating superior catalytic activity compared to MnCo_2_O_4_-BC (k_app_ = 0.0091 min^−1^, t_1/2_ = 77.9 min) and MnCo_2_O_4_-Neat (k_app_ = 0.0054 min^−1^, t_1/2_ = 128.3 min). These results highlight the role of stabilizers in enhancing photocatalytic performance by improving the material’s structural and electronic properties. The superior performance of MnCo_2_O_4_-CHT can be attributed to the strong interaction between chitosan and the MnCo_2_O_4_ framework, which enhances charge separation and reduces electron-hole recombination. Chitosan’s functional groups create additional reactive sites, facilitating efficient interaction with MB molecules. These findings align with previous studies showing that chitosan significantly improves the photocatalytic efficiency of metal oxides by modifying surface properties and reducing recombination rates. MnCo_2_O_4_-BC also shows improved catalytic performance compared to MnCo_2_O_4_-Neat, although its efficiency is lower than that of MnCo_2_O_4_-CHT. The biomass-cellulose stabilizer improves particle dispersion and introduces porosity, increasing the availability of active sites for the photocatalytic reaction. The templating effect of cellulose has been shown to enhance the structural integrity and porosity of spinel oxides, contributing to better adsorption and catalytic activity. In contrast, MnCo_2_O_4_-Neat exhibits the lowest catalytic efficiency due to the absence of stabilizers, resulting in less controlled morphology, lower surface area, and higher charge recombination rates. Previous reports have highlighted the limitations of unstabilized spinel oxides in achieving high catalytic performance, primarily due to structural irregularities and recombination of photogenerated carriers. To ensure that the observed methylene blue (MB) degradation resulted solely from photocatalytic activity rather than adsorption or photolysis, appropriate control experiments were performed under identical conditions. In the adsorption control test, the catalyst suspensions were stirred in the dark for 30 min to establish adsorption–desorption equilibrium prior to illumination. Only a negligible decrease (<5%) in MB concentration was detected, indicating minimal dye adsorption on the catalyst surface. In the photolysis control test, an MB solution without a catalyst was exposed to UV-rich solar light for 120 min, showing an insignificant change (<3%) in absorbance, confirming that direct photolysis of MB under UV-rich solar light was negligible. These results demonstrate that the observed dye degradation in the presence of MnCo_2_O_4_-based catalysts originates predominantly from photocatalytic reactions, validating the real contribution of the MnCo_2_O_4_ nanospinels and their biopolymer-stabilized composites to the degradation process.

In conclusion, the photocatalytic performance and kinetic analysis clearly demonstrate the significant impact of biodegradable stabilizers on MB degradation. MnCo_2_O_4_-CHT shows the most promising results due to its enhanced electronic and structural properties, followed by MnCo_2_O_4_-BC and MnCo_2_O_4_-Neat. These findings highlight the potential of stabilizer-assisted synthesis to optimize spinel oxides for efficient environmental remediation applications.

### 3.3. Photochemical Mechanism of MB Degradation

**Figure 11:**
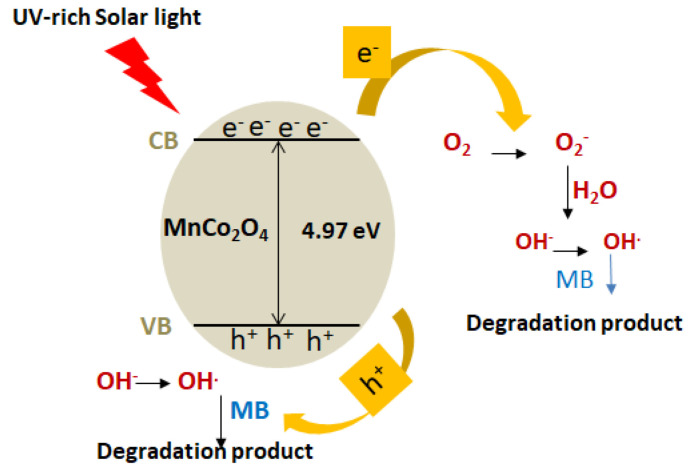
Proposed photocatalytic mechanism of methylene blue degradation over MnCo_2_O_4_–CHT under UV-rich solar irradiation.

The photocatalytic degradation of methylene blue (MB) using the MnCo_2_O_4_–CHT nanocomposite proceeds through a light-induced redox mechanism involving photoexcitation, charge separation, and the generation of reactive oxygen species (ROS). Upon irradiation with UV-rich solar light, photons with energy equal to or greater than the band gap energy (E_g_ = 4.97 eV) excite electrons (e^−^) from the valence band (VB) to the conduction band (CB) of MnCo_2_O_4_, leaving behind holes (h^+^) in the VB. The chitosan matrix, rich in amino (–NH_2_) and hydroxyl (^–^OH) functional groups, facilitates efficient charge transfer between the Mn and Co sites and suppresses electron–hole recombination through coordination and electron-donating interactions. The photogenerated electrons in the CB migrate to the catalyst surface, where they react with adsorbed O_2_ molecules to form superoxide radicals (^•^O_2_^−^). These radicals can undergo further protonation and transformation to yield hydroxyl radicals (^•^OH), powerful oxidizing species that decompose MB molecules into smaller, non-toxic intermediates and, ultimately, into CO_2_ and H_2_O. Simultaneously, the VB holes (h^+^) directly oxidize MB molecules or react with surface-adsorbed H_2_O or OH^−^ ions to generate additional ^•^OH radicals. The combined oxidative attack by ^•^O_2_^−^ and ^•^OH species, supported by the high surface area and charge-separation efficiency imparted by chitosan, results in rapid degradation of MB within 120 min. The following steps can represent the overall photocatalytic process (Figure 11):

1. Photon absorption and excitation:

MnCo_2_O_4_ + hν → e^−^(CB) + h^+^(VB)

2. Charge transfer and stabilization by chitosan:

Chitosan (–NH_2_/–OH) → acts as an electron donor/acceptor, suppressing e^−^–h^+^ recombination.

3. ROS generation:

e^−^ + O_2_ → ^•^O_2_^−^

^•^O_2_^−^ + H^+^ → HO_2_^•^ → H_2_O_2_ → ^•^OH

h^+^ + H_2_O/OH^−^ → ^•^OH

4. Pollutant degradation:

^•^OH/^•^O_2_^−^ + MB → Intermediates → CO_2_ + H_2_O

The chitosan’s functional groups enhance the catalyst’s surface charge distribution and electron transport, making MnCo_2_O_4_–CHT more efficient than unstabilized MnCo_2_O_4_. This synergistic effect between the spinel oxide and the biopolymer matrix is responsible for the 96% degradation efficiency observed.

Table 3 compares the photocatalytic performance of the MnCo_2_O_4_ nanospinels synthesized in this study with previously reported cobalt-based spinel photocatalysts used for methylene blue (MB) degradation [45,46,47,48,49,50,51]. The data highlight the significant enhancement achieved by incorporating biodegradable stabilizers—particularly chitosan (CHT), into the MnCo_2_O_4_ system. The MnCo_2_O_4_–CHT catalyst developed in this work achieved a 96% degradation efficiency within 120 min, with an apparent rate constant (k_app_ = 0.0203 min^−1^) and a band gap of 4.97 eV under UV-rich solar irradiation. This performance is comparable to or surpasses several previously reported cobalt spinel photocatalysts, including CoFe_2_O_4_ (99% in 60 min, 2.2–2.3 eV) [46], and Co_x_Ni_1−x_Fe_2_O_4_ (92% in 100 min, 2.2–2.32 eV) [48]. Although these materials operate under visible light due to their narrower band gaps, the MnCo_2_O_4_–CHT catalyst demonstrates exceptional efficiency even under UV-rich conditions without requiring dopants or co-catalysts. The superior performance of MnCo_2_O_4_–CHT can be attributed to the synergistic effect of chitosan, whose amino (–NH_2_) and hydroxyl (–OH) functional groups enhance metal-ion coordination, charge separation, and ROS generation, thereby enabling more effective photocatalytic reactions. In comparison, MnCo_2_O_4_–BC achieved 65% degradation (k_app_ = 0.0091 min^−1^), confirming the beneficial but less pronounced effect of cellulose as a stabilizer. The unmodified MnCo_2_O_4_–Neat exhibited only 45% degradation, highlighting the critical role of stabilizers in tailoring the surface and electronic properties to improve activity. While some reported materials such as MnCo_2_O_4_._5_ NPs [45] achieve complete degradation using chemical oxidants like peroxymonosulfate (PMS), the MnCo_2_O_4_–CHT catalyst attains comparable performance without external oxidants, demonstrating a greener and more sustainable photocatalytic route. Moreover, despite its relatively larger band gap, MnCo_2_O_4_–CHT maintains high activity due to enhanced surface reactivity and efficient electron–hole separation facilitated by chitosan stabilization. Overall, the comparison confirms that stabilizer-assisted MnCo_2_O_4_ nanospinels, particularly those synthesized with chitosan, offer a promising alternative to conventional doped or composite cobalt spinels. Their high degradation rate, excellent stability, and environmentally friendly synthesis approach make them viable candidates for practical wastewater treatment and large-scale photocatalytic applications.

## 4. Conclusions

This study highlights the successful synthesis of MnCo_2_O_4_ nanospinels using biodegradable stabilizers—chitosan (CHT) and biomass-cellulose (BC)—and their enhanced photocatalytic performance for methylene blue (MB) degradation. MnCo_2_O_4_-CHT exhibited the highest efficiency (96% MB degradation in 120 min) with the highest rate constant (k_app_ = 0.0203 min^−1^) and shortest half-life (t_1/2_ = 34.2 min), followed by MnCo_2_O_4_-BC (65%) and MnCo_2_O_4_-Neat (45%). The stabilizers improved crystallinity, porosity, and charge separation, significantly enhancing photocatalytic activity. These findings demonstrate the potential of stabilizer-assisted MnCo_2_O_4_ nanospinels as sustainable and efficient photocatalysts for environmental remediation, paving the way for eco-friendly wastewater treatment solutions.

## Figures and Tables

**Table 1:** Textural characteristics of the synthesized MnFe_2_O_4_ synthesized using various biodegradable stabilizers.

Samples	S_BET_/m^2^ g^−1^	d_BJH_/nm	^v^_BJH_/cm^3^ g^−1^
MnCo_2_O_4_-Neat	33.44	26.2	0.0024
MnCo_2_O_4_-CHT	27.42	29.1	0.0019
MnCo_2_O_4_-BC	29.32	27.3	0.0013

**Table 2:** Kinetic parameters (k_app_ and t_1_/_2_) and the degradation performance (%) of MB MnCo_2_O_4_-Neat, MnCo_2_O_4_-BC, and MnCo_2_O_4_-CHT nanospinel at pH (7) after 120 min of UV-rich solar irradiation at pH of 7.

Samples	k_app_, min^−1^	t_1/2_	R^2^	Degradation (%)
MnCo_2_O_4_-Neat	0.0054	128.3	0.9997	45%
MnCo_2_O_4_-BC	0.0091	77.9	0.9986	65%
MnCo_2_O_4_-CHT	0.0203	34.2	0.9997	96%

**Table 3:** Comparative photocatalytic performance of MnCo_2_O_4_ nanospinels synthesized in this study and previously reported cobalt-based spinel photocatalysts for methylene blue (MB) degradation, including band gap energies and reaction conditions.

Photocatalyst	Light Source/Conditions	MB Degradation (%)	Time (min)	Rate Constant (k_app_, min^−1^)	Band Gap (eV)	Ref.
MnCo_2_O_4_–CHT	UV-rich solar simulator (AM 1.5G, 100 mW/cm^2^), pH 7	96%	120	0.0203	4.97	This work
MnCo_2_O_4_._5_ NPs	Ambient light	100%	25	—	—	[45]
CoFe_2_O_4_ NPs	UV irradiation, pH 5–7	99%	60	—	2.2–2.3	[46]
Al-doped/CoFe_2_O_4_	Visible light (200 W), pH 11	93%	120	—	—	[47]
Co_x_Ni_1−x_Fe_2_O_4_	Direct visible light	92%	100	—	2.20–2.32	[48]
Dy^3+^-doped Ni–Co ferrite/rGO	Visible light	73%	120	—	2.27	[49]
Li–Co ferrite (Bi-doped)	UV–Visible light	93.8%	90	0.0203	2.70–3.95	[50]
Co_3_O_4_ (Green-synthesized)	Visible light, neutral pH	93%	175	—	2.00	[51]

## Data Availability

The original contributions presented in this study are included in this article. Further inquiries can be directed to the corresponding author.
